# Induced CNS expression of CXCL1 augments neurologic disease in a murine model of multiple sclerosis via enhanced neutrophil recruitment

**DOI:** 10.1002/eji.201747442

**Published:** 2018-05-16

**Authors:** Jonathan J. Grist, Brett S. Marro, Dominic D. Skinner, Amber R. Syage, Colleen Worne, Daniel J. Doty, Robert S. Fujinami, Thomas E. Lane

**Affiliations:** ^1^ Department of Pathology Division of Microbiology and Immunology University of Utah School of Medicine Salt Lake City UT USA; ^2^ Immunology Inflammation and Infectious Disease Initiative University of Utah UT USA; ^3^ Department of Molecular Biology and Biochemistry University of California Irvine CA USA

**Keywords:** Autoimmunity, Chemokines, Demyelination, Neuroinflammation, Neutrophils

## Abstract

Increasing evidence points to an important role for neutrophils in participating in the pathogenesis of the human demyelinating disease MS and the animal model EAE. Therefore, a better understanding of the signals controlling migration of neutrophils as well as evaluating the role of these cells in demyelination is important to define cellular components that contribute to disease in MS patients. In this study, we examined the functional role of the chemokine CXCL1 in contributing to neuroinflammation and demyelination in EAE. Using transgenic mice in which expression of CXCL1 is under the control of a tetracycline‐inducible promoter active within glial fibrillary acidic protein‐positive cells, we have shown that sustained CXCL1 expression within the CNS increased the severity of clinical and histologic disease that was independent of an increase in the frequency of encephalitogenic Th1 and Th17 cells. Rather, disease was associated with enhanced recruitment of CD11b^+^Ly6G^+^ neutrophils into the spinal cord. Targeting neutrophils resulted in a reduction in demyelination arguing for a role for these cells in myelin damage. Collectively, these findings emphasize that CXCL1‐mediated attraction of neutrophils into the CNS augments demyelination suggesting that this signaling pathway may offer new targets for therapeutic intervention.

## Introduction

EAE is a T cell‐driven autoimmune disease sharing many clinical and pathologic features with the human demyelinating disease MS 1, 2, 3. In EAE, CD4^+^ and CD8^+^ T‐cells specific for self‐antigens expressed in CNS myelin initiate a localized inflammatory process that results in demyelination, axonopathy, and clinical deficits 4, 5, 6. The concept that the adaptive immune response is critical for new lesion development and disease progression in MS is emphasized in that FDA‐approved disease modifying therapies are designed to limit infiltration of encephalitogenic lymphocytes into the CNS 7. Among the mechanisms by which myelin‐reactive CD4^+^ T‐cells contribute to disease is through localized secretion of cytokines, e.g. IL‐17 that increases expression of chemokines that attract myeloid cells into the CNS 8. Monocytes and macrophages amplify white matter damage through active stripping of the myelin sheath leading to axonal damage, presentation of novel myelin epitopes to T cells and amplifying neuroinflammation through secretion of proinflammatory molecules. In addition, IL‐17 secretion increases expression of the neutrophil chemoattractants CXCL1 and CXCL2 resulting in recruitment and accumulation of these cells within the CNS 9.

Increasing evidence in both preclinical models of MS as well as from MS patients have highlighted a potentially important role for neutrophils in demyelination 10, 11, 12, 13, 14, 15, 16, 17, 18, 19, 20. Neutrophil depletion delays the onset of clinical symptoms in EAE mice arguing for a role for these cells in disease initiation and/or lesion formation 9, 13, 17, 19. Studies designed to decipher the mechanisms by which neutrophils augment disease progression point to increases in vascular permeability as well as secretion of reactive nitrogen and oxygen species 14, 18. In patients with relapsing‐remitting MS, there are elevated systemic levels of neutrophil‐activating chemokines including CXCL1, CXCL5, and neutrophil elastase accompanied by increased numbers of neutrophils having a primed phenotype 10, 12. Collectively, these findings from preclinical models as well as MS patients argue for a role for neutrophils in contributing to disease.

Given the transient nature of neutrophils in terms of having a short half‐life, limited presence within lesions, and tightly regulated expression of chemotactic signals underscores the challenging aspects involved in understanding the role of these cells in autoimmune demyelination. To overcome some of these limitations, we have derived a transgenic mouse whereby expression of CXCL1 is under the control of a tetracycline‐inducible promoter that is active within glial fibrillary acidic protein (GFAP)‐positive astrocytes 21. Recent studies have emphasized an important role for CXCL1 and it is receptor CXCR2 during the effector phase of EAE 9, 22, 23, 24. Although resident cells of the CNS including microglia and endothelial cells express CXCL1 24, 25, astrocytes have been shown to express CXCL1 in EAE as well as in MS lesions 26, 27, 28 and this informed our decision to select astrocytes as our target cell for ectopic expression of CXCL1. Our present study was undertaken to better understand how elevated and sustained neutrophil recruitment into the CNS impacts EAE disease progression and white matter damage. Our findings indicate that increased expression of CXCL1 within the CNS results in more severe EAE as measured by both clinical disease and demyelination. The CXCL1‐mediated disease enhancement was not dependent on an increase in myelin‐reactive Th1 or Th17 cells but correlated with increased numbers of neutrophil infiltration into the spinal cord. Indeed, depletion of neutrophils resulted in a reduction in the severity of white matter damage highlighting a role for these cells in demyelination.

## Results

### Increased clinical disease following Dox‐induced expression of CXCL1

**Figure 1:**
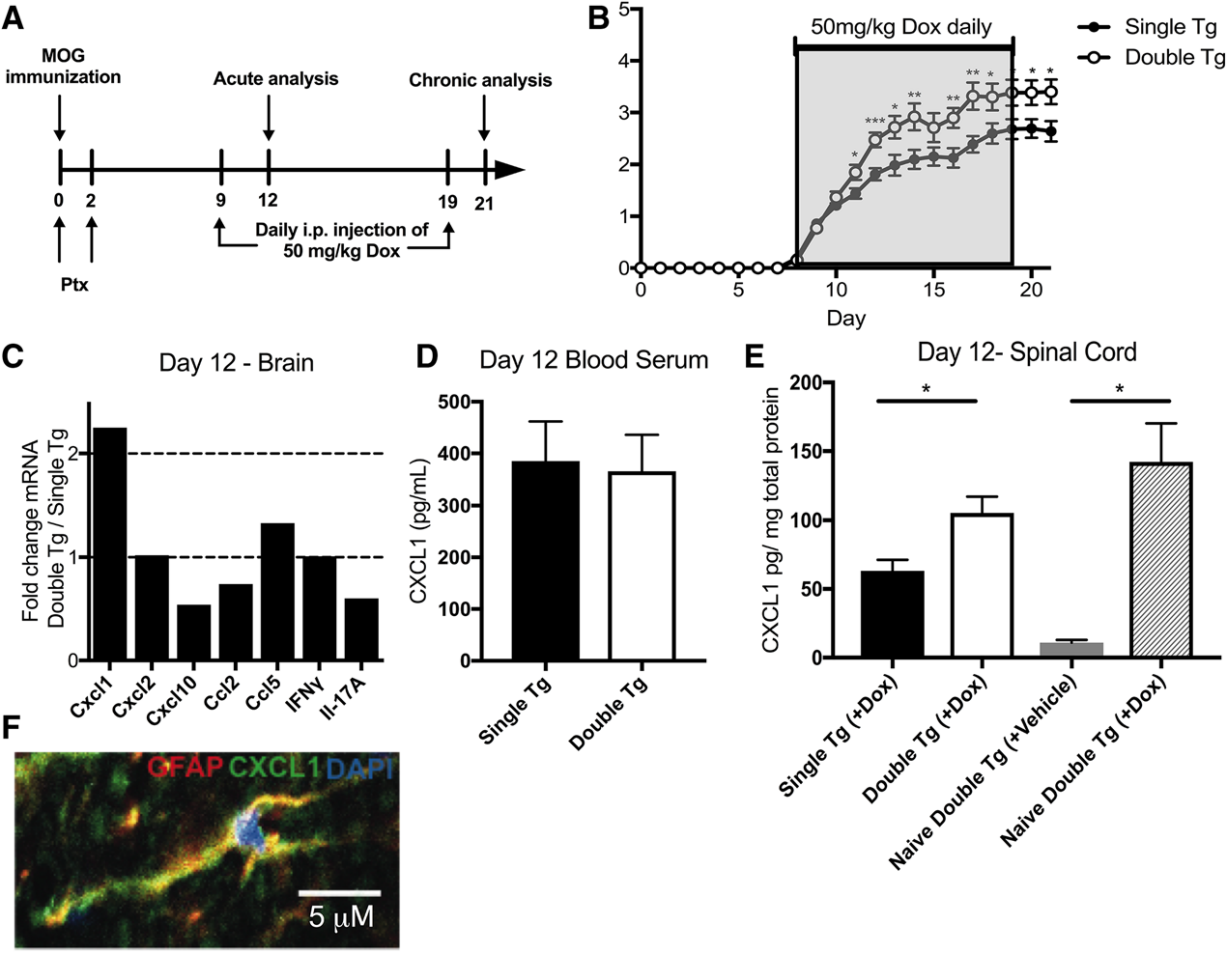
Dox‐induced CXCL1 expression increases clinical disease severity following MOG_35–55_‐induced EAE. (A) Schematic outline of experimental approach for disease induction and Dox administration. MOG, MOG_35–55_ peptide; PTX, pertussis toxin. (B) Clinical disease score was measured in MOG_35–55_‐immunized double tg mice (*n*  =  26) compared to single tg (*n*  =  29) following Dox treatment. Data were pooled from four independent experiments with between 6–8 mice per experiment. (C) mRNA transcripts of *Cxcl1* and other pro‐inflammatory cytokines/chemokines in Dox‐treated double tg mice (*n*  =  2) compared to single tg mice (*n*  =  2) at day 12 p.i; data presented is representative of two independent experiments with a total of four mice per experimental group. CXCL1 levels in serum (D) and spinal cords (E) of MOG_35–55_‐immunized mice was determined by ELISA's at day 12 postimmunization. CXCL1 expression in spinal cords of either Dox‐ or vehicle‐treated double tg mice in the absence of peptide immunization (naïve) was also determined 3 days following last Dox injection (E); data is pooled from two independent experiments with between 2–4 mice for each experiment. (F) Representative immunofluorescence staining (60X) showing colocalization of CXCL1 protein (green) with GFAP‐positive (red) astrocytes in double tg mice at day 12 following Dox treatment (scale bar = 5 μm). Statistical analysis employed unpaired two‐tailed Student's *t* test; data presented as average ± SEM. *p < 0.05; **p < 0.01; ***p < 0.001.

Following induction of EAE, CD4^+^ T‐cell‐derived IL‐17 expression in the CNS potentiates disease by enhancing expression of the neutrophil chemoattractants CXCL1 and CXCL2 9. The accumulation of neutrophils in the CNS is rapid yet transient and reflects the expression kinetics of CXCL1 and CXCL2. To better understand how neutrophil infiltration impacts demyelination and disease progression in EAE, transgenic (tg) mice were engineered to express CXCL1 within GFAP‐positive astrocytes upon doxycycline administration, as previous studies have shown these cells to be the primary source of CXCL1 in models of neuroinflammation 29, 30. Doxycyline (Dox) responsive double tg mice (pBI‐CXCL1‐rtTA) or single tg controls lacking the reverse tetracycline transactivator (rtTA) were immunized with MOG_35–55_ peptide and treated daily with Dox (50 mg/kg) i.p. between days 9 through 19 postimmunization (Fig. 1A). Onset of clinical disease was similar between Dox‐treated double tg and single tg mice (day 9 p.i.), but double tg mice exhibited a significant increase in clinical disease severity compared to single tg mice (Fig. 1B). Examination of mRNA transcripts encoding proinflammatory chemokines/cytokines indicated a selective >twofold increase in CXCL1 in Dox‐treated double tg mice versus single tg mice at day 12 postimmunization that represents a time in which there is a separation in clinical disease between double and single tg mice following MOG_35–55_ immunization. No differences in expression of transcripts encoding CXCL2, CXCL10, CCL2, CCL5, IFN‐γ, and IL‐17A at day 12 between Dox‐treated double tg and single tg mice were observed (Fig. 1C). A significant (*p* < 0.05) increase in CXCL1 protein was detected only in the spinal cord (Fig. 1E) and not in the serum (Fig. 1D) of double tg mice 12 days postimmunization compared to single tg controls. Notably, we also observed a significant (*p* < 0.05) increase in CXCL1 protein within the spinal cords of Dox‐treated naïve mice in which EAE was not induced indicating that disease induction did not selectively enhance transgene expression (Fig. 1E). We did not detect differences in proteins levels of G‐CSF, GM‐CSF, or IL‐17 (data not shown) either prior to or following Dox treatment of double tg or single tg EAE mice. Immunofluorescence staining confirmed astrocytes (GFAP+) as the primary cellular source of CXCL1 in Dox‐treated double tg EAE mice (Fig. 1F). Together, these findings indicate that CXCL1 expression was specifically elevated in the CNS of Dox‐treated double tg mice that correlated with an increase in clinical disease.

### Induced expression of CXCL1 does not increase either T cell or macrophage infiltration into the CNS

**Figure 2:**
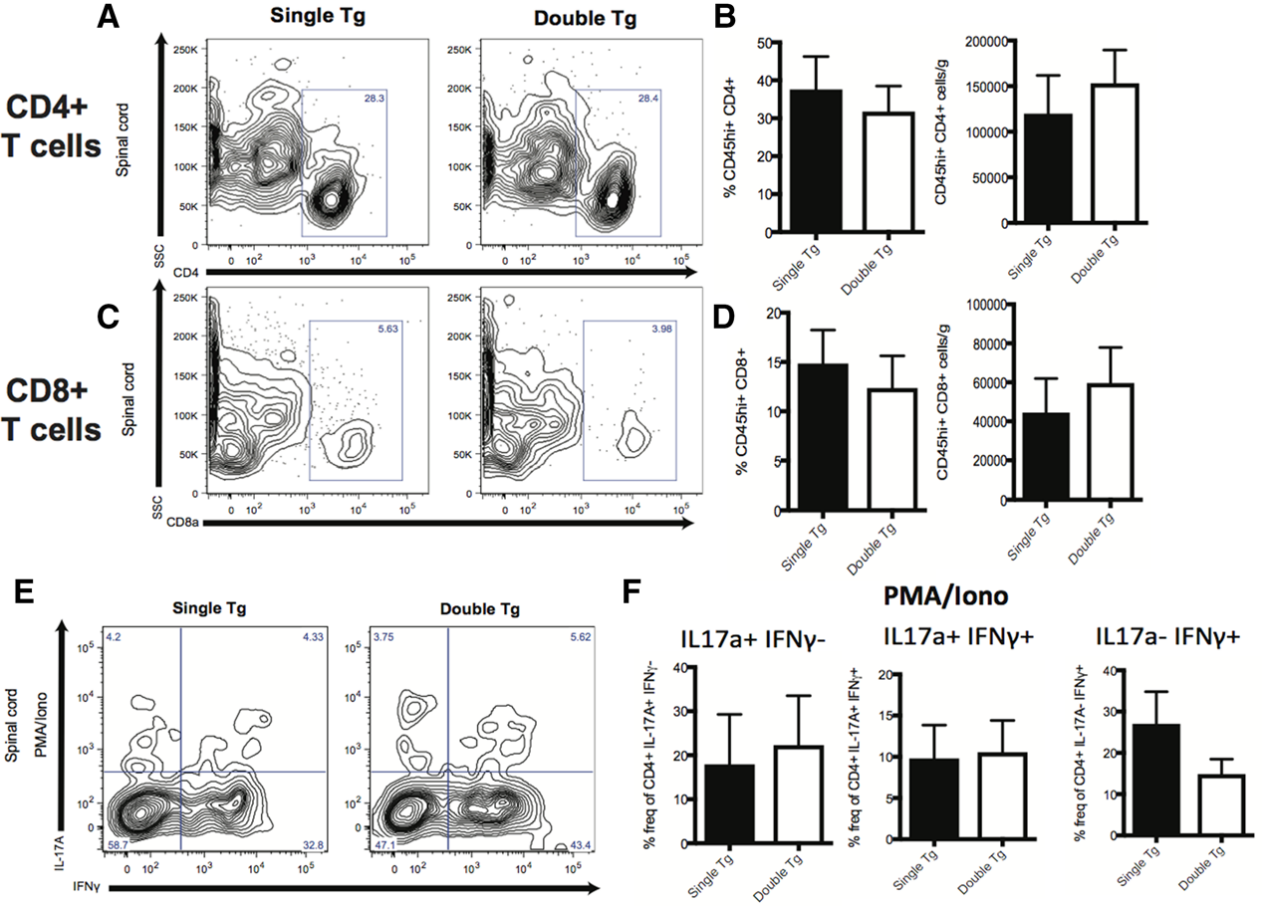
T‐cell infiltration into the CNS is not altered following Dox‐induced expression of CXCL1. Spinal cords were removed at day 12 following MOG_35–55_ immunization of Dox‐treated double tg (*n*  =  10) and single tg (*n*  =  10) mice and the presence of CD45^+^CD4^+^ T cells (A, B) or CD45^+^CD8a^+^ T cells (C, D) determined by flow cytometry. The gating scheme employed is depicted in Supporting Information Fig. S1. Representative contour blots from experimental animals are shown in panels *A* and *C*. Data in panels *B* and *D* are pooled from two independent experiments with a minimum of three mice per experimental group; data is presented as mean ± SEM. (E and F) Expression of cytokines IL‐17A and/or IFN‐γ in Dox‐treated double tg or single tg following PMA/ionomycin treatment of CD4^+^ T‐cells isolated from spinal cords at day 12 following MOG_35–55_ immunization. Data in panel *E* are representative contour plots showing the results of intracellular staining for IL‐17A and IFN‐γ following PMA/ionomycin treatment of CD4^+^ T cells. The gating scheme employed is depicted in Supporting Information Fig. S2. Data in panel *F* represent quantification of intracellular cytokine staining and pooled from three independent experiments with a minimum of two mice per experimental group and presented as mean ± SEM and statistical analysis employed unpaired two‐tailed Student's *t* test.

**Figure 3:**
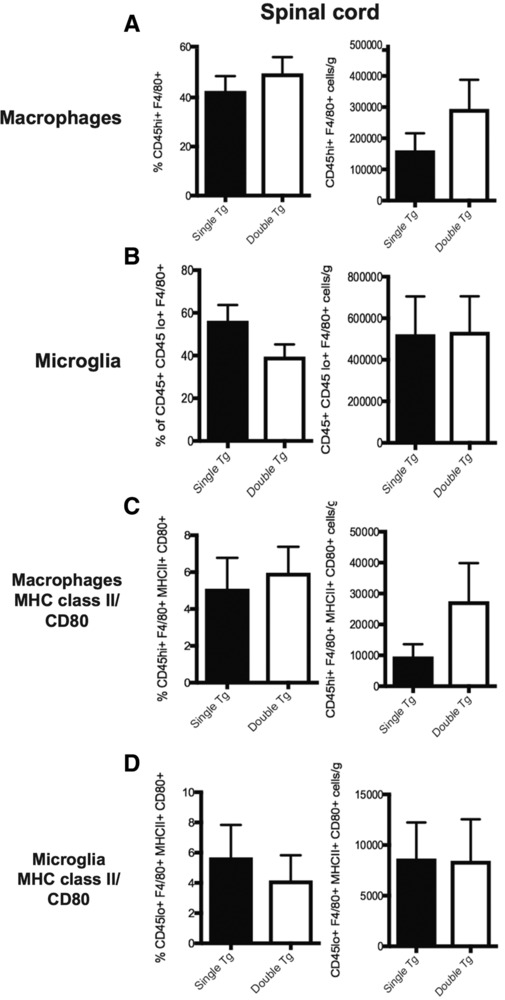
Dox‐induced CXCL1 within the CNS does not increase microglia/macrophage activation. Dox‐treated double and single tg mice were sacrificed at day 12 following MOG_35–55_ immunization and spinal cords removed to assess macrophage (CD45^hi^ F4/80^+^) (A) and microglia (CD45^lo^F4/80^+^) (B) activation via flow cytometric staining between double tg (*n*  =  10) or single tg (*n*  =  10) mice. The gating scheme employed is depicted in Supporting Information Fig. S3. Surface expression of activation markers MHC class II and CD80 was also examined on macrophages (C) or microglia (D) between double tg (*n*  =  7) and single tg (*n*  =  7) mice. Data in panels *A* and *B* were pooled from three independent experiments; data in panels *C* and *D* from two independent experiments. Data are presented as mean ± SEM and statistical analysis employed unpaired two‐tailed Student's *t* test.

MOG_35–55_‐induced EAE disease correlates with CNS infiltration of Th1 and Th17 CD4^+^ T‐cells reactive against the encephalitogenic MOG peptide 31. In addition, other immune subsets including CD8^+^ T cells, B cells, and macrophages are also considered important in amplifying clinical disease and pathology 31. We determined if overexpression of CXCL1 in the CNS of Dox‐treated double tg mice immunized with MOG_35–55_ peptide altered the infiltration of inflammatory leukocytes into the CNS. We did not detect differences in the frequency or numbers of CD45^hi^ leukocytes (data not shown), CD4^+^ T cells (Fig. 2A and B), or CD8^+^ T cells (Fig. 2C and D) in spinal cords of Dox‐treated double tg mice compared to single tg mice. Moreover, there was no difference in intracellular CD4^+^ T‐cell cytokine expression of IFN‐γ, IL‐17A, or coexpressing IFN‐γ and IL‐17A within the spinal cords at day 12 p.i. following PMA/ionomycin stimulation (Fig. 2E and F). We did not observe changes in macrophages (CD45^hi^F4/80^+^) (Fig. 3A), microglia (CD45^lo^F4/80^+^) (Fig. 3B), or macrophages or microglia coexpressing MHC class II and CD80 (Fig. 3C and D) of Dox‐treated double and single tg EAE mice. Collectively, these findings argue that the increase in clinical disease following Dox‐induced CXCL1 expression within the CNS is not due to increased infiltration of myelin‐reactive CD4^+^ T cells nor other immune cells that are known to contribute to EAE disease severity.

### Increased neutrophil accumulation in response to Dox‐induced expression of CXCL1

**Figure 4:**
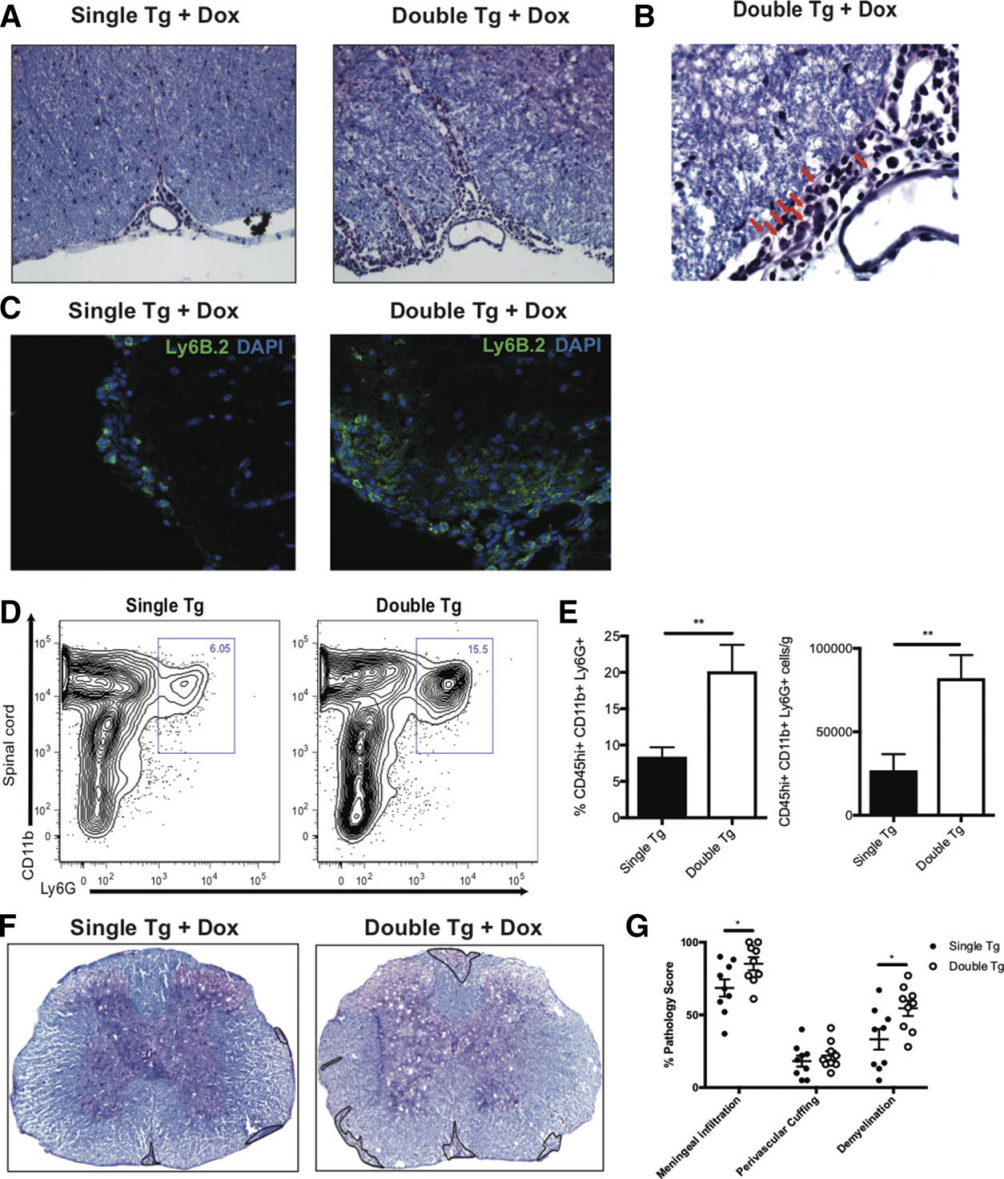
Neutrophil infiltration into the CNS is associated with increased demyelination. MOG_35–55_‐immunized double and single tg mice treated with Dox were sacrificed at day 12 postimmunization and spinal cords removed to assess histopathology and myeloid cell infiltration. (A) Representative H&E/Luxol fast blue staining of the spinal cords of experimental mice revealed increased inflammation within the anterior median fissure of double tg mice compared to single tg mice, 20X magnification. (B) Representative image of a spinal cord of Dox‐treated double tg mouse showing cells with multilobed nuclei (red arrows) characteristic of neutrophils, 60X magnification. (C) Representative immunofluorescent staining for the neutrophil‐specific surface antigen Ly6B.2 of double tg mice compared to single tg mice, 20X magnification. (D) Representative contour flow graphs showing increase in neutrophils (CD45^hi^ CD11b^+^ Ly6G^+^) within the spinal cords of double tg mice compared to single tg mice. The gating scheme employed is depicted in Supporting Information Fig. S4. (E) Quantification of neutrophil flow staining in the spinal cords of double tg mice (*n* = 10) compared to single tg mice (*n*  =  10). Data in panel *E* were derived from three independent experiments and data presented as mean ± SEM. (F) Representative H&E/LFB stained spinal cord images (4X) demonstrated increased demyelination (outlined in black) in double tg mice compared to single tg mice at day 21 p.i. (G) Quantification of neuropathology indicated increased meningeal inflammation (*p* < 0.05), perivascular cuffing and demyelination (*p* < 0.05) at score at day 21 p.i. in double tg mice (*n* = 9) compared to single tg mice (*n*  =  9); data pooled from three independent experiments. Statistical analysis employed unpaired two‐tailed Student's *t* test, **p < 0.01.

CXCL1 is a potent chemoattractant of neutrophils expressing the chemokine receptor CXCR2 and its overexpression from astrocytes enhances the migration and accumulation of neutrophils in the white matter tracts of the spinal cords in a model of viral‐induced encephalomyelitis 21, 32. We next investigated whether Dox‐induced overexpression of CXCL1 promotes neutrophil accumulation in double tg mice induced with EAE. Histopathologic examination of spinal cord sections revealed increased localization of inflammatory cells in the anterior median fissure and meninges of the spinal cords of Dox‐treated double tg mice compared to single tg EAE mice (Fig. 4A). The majority of inflammatory cells within spinal cords of double tg mice had a multilobed nucleus characteristic of neutrophils (Fig. 4B). Immunofluorescence staining revealed increased numbers of cells positive for the neutrophil‐associated surface antigen Ly6B.2 within the spinal cords of Dox‐treated double tg EAE mice compared to single tg mice treated with Dox (Fig. 4C). In addition, flow analysis of spinal cords isolated from experimental mice indicated the frequency and numbers of neutrophils (CD45^hi^CD11b^+^Ly6G^+^) within the spinal cords of Dox‐treated double tg mice were significantly (*p* < 0.01) increased compared to single tg mice (Fig. 4D and E). Importantly, we did not observe an increase in Ly6C+ monocytes in either brain or spinal cords of double tg mice treated with Dox (data not shown). Together, these findings argue that the Dox‐induced increase in CXCL1 expression within the CNS enhances the recruitment and accumulation of neutrophils within the spinal cords associated increased clinical disease severity. Using a model of viral‐induced demyelination, we have previously shown that Dox‐induced CXCL1 expression within the CNS increases the severity of white matter damage and this was correlated with enhanced neutrophil recruitment into the spinal cord 21. Assessment of spinal cord pathology between Dox‐treated single tg and double tg mice was assessed using H&E/LFB staining at day 21 p.i. (Fig. 4F). We recorded an increase in both meningeal infiltration (*p* < 0.05) and demyelination (*p* < 0.05) of double tg compared to single tg was observed (Fig. 4G).

### Neutrophils increase the severity of demyelination in Dox‐treated double tg EAE mice

**Figure 5:**
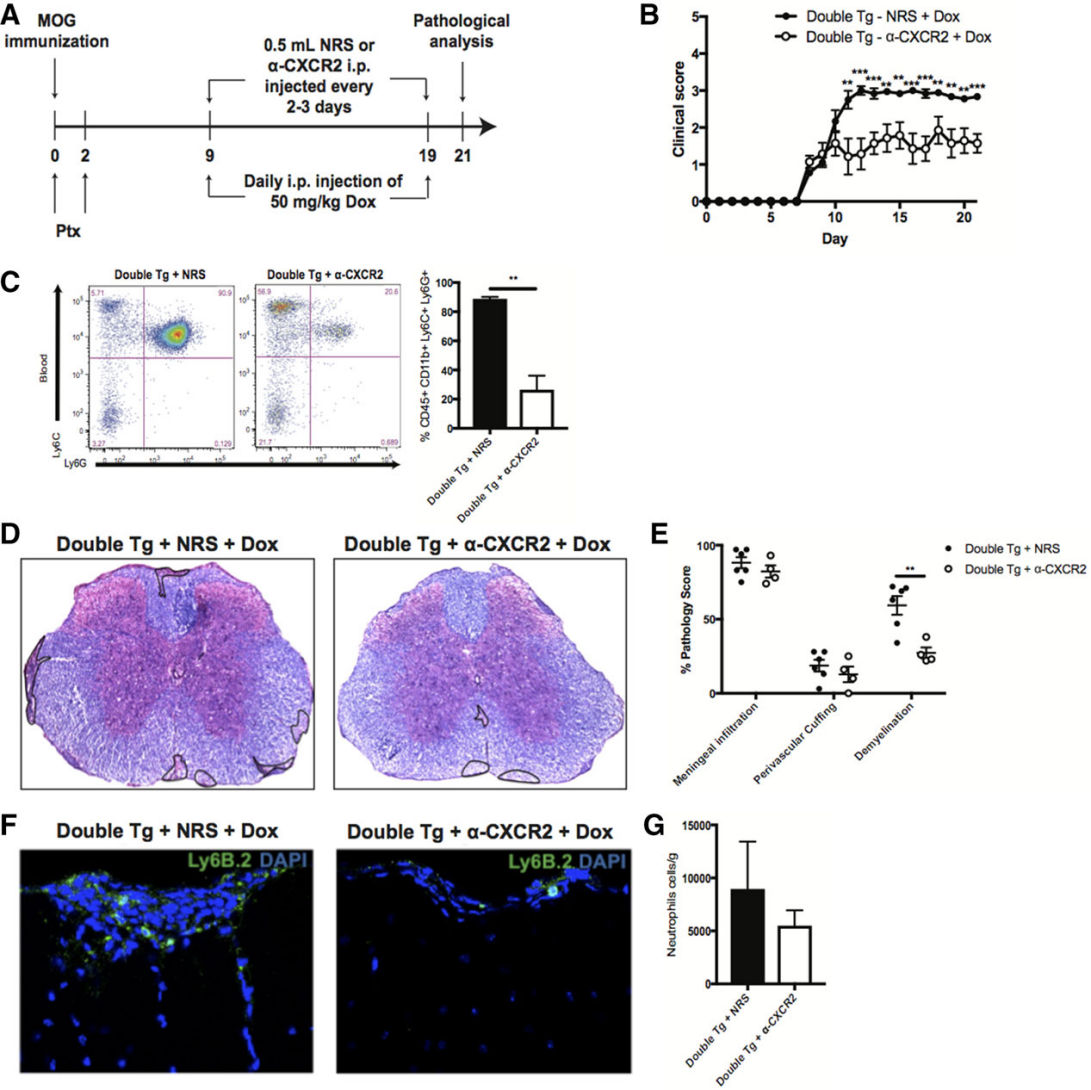
Targeting neutrophils diminishes the severity of demyelination. MOG_35–55_‐immunized double and single tg mice treated with Dox were sacrificed at day 21 postimmunization and spinal cords removed to assess histopathology and myeloid cell infiltration. (A) Schematic outline for experimental design to target neutrophils in Dox‐treated double tg mice via injection of either anti‐CXCR2 or NRS control antibody. (B) Treatment with anti‐CXCR2 in Dox‐treated double tg mice (*n*  =  7) compared to animals treated with NRS (*n*  =  9); data pooled from three independent experiments with 2–3 mice per experiment. (C) Anti‐CXCR2 treatment in Dox‐treated double tg mice (*n*  =  3) resulted in reduced (*p* < 0.01) levels of neutrophils (CD45^hi^ CD11b^+^ Ly6C^+^ Ly6G^+^) in blood compared to NRS‐treated mice (*n*  =  3); data derived from two independent experiments presented as mean ± SEM. The gating scheme employed is depicted in Supporting Information Fig. S5. (D) Representative H&E/LFB images depicting a reduction in demyelination (outlined in black) in Dox‐treated double tg compared to single tg mice and (E) quantification showed a significant (*p* < 0.01) reduction in demyelination in anti‐CXCR2 treated mice (*n*  =  6) compared to NRS‐treated mice (*n*  =  4); data is representative of two independent experiments. (F) Representative immunofluorescent staining for neutrophils (Ly6B.2) reveals fewer positive cells within the spinal cords of anti‐CXCR2‐treated mice compared to NRS‐treated mice. (G) Quantification of spinal cord Ly6B.2‐positive neutrophils reveals reduced numbers in anti‐CXCR2‐treated mice (*n*  =  3) compared to NRS‐treated mice (*n*  =  3); data derived from two independent experiments presented as mean ± SEM. **p < 0.01, ***p < 0.001.

In order to determine whether neutrophils contributed to enhancement of demyelination, MOG_35–55_‐immunized double tg mice treated with Dox were administered anti‐CXCR2 antisera which we have previously shown to effectively target neutrophils 30, 32. Anti‐CXCR2 or NRS was administered to Dox‐treated double tg mice at defined times following EAE induction (Fig. 5A). Administration of anti‐CXCR2 antisera to Dox‐treated double tg mice resulted in a dramatic reduction in clinical disease severity compared to animals treated with NRS alone (Fig. 5B) and this was correlated with reduced numbers of circulating CD45^hi^CD11b^+^Ly6C^+^Ly6G^+^ neutrophils (Fig. 5C). The reduction in clinical disease in Dox‐treated double tg mice treated with anti‐CXCR2 correlated with a significant (*p* < 0.01) reduction in the severity of demyelination compared to NRS‐treated mice (Fig. 5D and E) and this was associated with reduced numbers of neutrophils present within the spinal cords (Fig. 5F and G). Together, these findings indicate that neutrophils can enhance the severity of demyelination in MOG_35–55_‐induced EAE.

## Discussion

MS is characterized by CNS inflammatory lesions consisting of activated lymphocytes and monocyte/macrophages that are associated with demyelinating lesions within the brain and spinal cord 24, 33. The importance of these cells to disease is highlighted in that FDA‐approved disease modifying therapies used in treatment of relapsing remitting MS limit immune cell infiltration and lesion development 34, 35. Nonetheless, there is increasing interest in the potential role of other cell types, including neutrophils, in participating in demyelination. The diverse functions of neutrophils include phagocytosis, release of toxic granules, and secretion of ROS, nitrogen species, and neutrophil extracellular traps that can lead to bacterial clearance and tissue damage 36, 37, 38, 39. In addition, neutrophils can produce many different cytokines and chemokines that participate in tailoring the immune response as well as enhancing neuroinflammation 36, 37, 38, 39. Although there is direct evidence that neutrophils are important in both disease initiation and progression in EAE models only indirect evidence exists showing these cells also play a role in MS pathogenesis 40. Since neutrophils are typically short‐lived and have a half‐life in circulation (between 6 and 8 hours in humans) 41, direct evidence of neutrophils in MS lesions from available autopsy tissue samples has been limited. Nonetheless, recent studies from Segal and colleagues 12 have demonstrated increased systemic levels of the chemokines CXCL1 and CXCL5 as well as neutrophil elastase that correlated with lesion burden and clinical disability in MS patients arguing for a role for neutrophils in augmenting disease.

The current study was performed to better understand the functional role of CXCL1 within the CNS following induction of MOG_35–55_ immunized EAE in mice. CXCL1 is a member of the ELR (+) family of CXC chemokines and binds with high affinity to the chemokine receptor CXCR2 11, 42. Neutrophils express CXCR2 and signaling enhances egress out of the BM and aids in homing to sites of inflammation 36. In the context of EAE, CXCL1 is detected early in the CNS 24 both prior to and following disease onset 12. Mice treated with anti‐CXCL1 blocking antibody showed a delayed onset of disease and decreased clinical disease correlating with diminished numbers of Ly6G^+^ neutrophils yet CNS T‐cell infiltration was not affected arguing for a role for neutrophils in disease 24. Similarly, a better understanding of molecules, e.g. CXCL1 that influence neutrophil accumulation within the CNS also allows insight into the therapeutic relevance of these potential targets. Lira et al. 43 have previously shown that transgenic mice in which CXCL1 was constitutively expressed from oligodendrocytes led to the development of neurologic disease associated with microglia and astrocyte reactivity that correlated with increase in neutrophil accumulation within the brain. Using the animals described in this study in which transgenic *Cxcl1* is selectively expressed in astrocytes upon Doxycycline treatment, we recently showed that increased and sustained expression of CXCL1 was associated with increased clinical disease and demyelination in animals infected with the neurotropic JHM strain of mouse hepatitis virus (JHMV) 21. Enhanced clinical and histologic disease correlated with a dramatic increase in neutrophil infiltration into the CNS; targeted ablation of neutrophils resulted in a reduction in the severity of demyelination further supporting an important role for neutrophils in augmenting white matter damage 21.

Using this transgenic mouse in which CXCL1 expression is selectively induced within astrocytes allows for an increased understanding of how neutrophils may augment neuroinflammation and demyelination in EAE. Similar to our previous report using JHMV infection of CXCL1 double transgenic mice 21, we found that Dox administration resulted in elevated CXCL1 transgene expression within the brains and spinal cords of double tg mice that was restricted to astrocytes and correlated with rapid and robust neutrophil migration to the CNS compared to Dox‐treated controls. As a result of CXCL1 transgene expression, MOG_35–55_‐immunized double tg mice displayed sustained increase in clinical disease severity. Increased accumulation of neutrophils in the CNS did not affect the expression of proinflammatory factors that could potentially attract inflammatory leukocytes and/or increase numbers of resident glial cells. Indeed, we observed no statistical difference in the frequencies or numbers of either activated macrophages or microglia expressing MHC class II and CD80 between Dox‐treated double tg and single tg mice. Furthermore, we did not detect any increase in either frequency or numbers of CNS inflammatory CD4^+^ or CD8^+^ T cells in Dox‐treated double tg mice compared to Dox‐treated single tg mice. In addition, there were no differences in the expression of IFN‐γ and/or IL‐17A by CD4^+^ T‐cells between Dox‐treated double and single tg mice. Collectively, these findings indicate that the increase in MOG_35–55_‐induced clinical disease upon Dox‐induced CXCL1 expression is not the result of increased infiltration/activation of either macrophages/microglia or T cells. Of additional interest is our observation that Dox‐treatment of naïve double tg mice resulted in increased expression of CXCL1 within the CNS and these findings argue that induction of EAE is dispensable in terms increased expression of this transgene. Ongoing studies in our laboratory are determining if sustained CXCL1 expression within the CNS is sufficient to contribute to demyelination mediated by neutrophil accumulation in the absence of MOG_35–55_ immunization.

In contrast with our findings, Omari et al. 44 demonstrated that overexpression of CXCL1 by astrocytes protected animals from EAE by limiting neuroinflammation as well as increasing numbers of oligodendrocytes associated with enhanced remyelination. This difference in results most likely reflects that the *Cxcl1* transgene employed in our study was lacking the 3’ UTR resulting in an increase in the half‐life of CXCL1 mRNA and therefore sustained CXCL1 production and subsequently neutrophil accumulation whereas Omari et al. was using a transgene containing the 3’UTR and nonsustained expression of CXCL1 44. We do not believe CXCL1 had any direct cytotoxic activity on oligodendroglia as we have previous shown that treatment of differentiating mouse oligodendrocyte progenitor cells with recombinant CXCL1 does not kill these cells 45. Rather, our findings argue that increased white matter damage is associated with a selective increase in neutrophil accumulation within the CNS and this is consistent with earlier studies from our laboratory using the CXCL1 transgenic animals in a model of viral‐induced neurologic disease 21. Although recent studies suggest that Ly‐6C+ myeloid precursor cells play a pathogenic role during autoimmune demyelination 46, our findings reveal that the increase in tissue damage was not the result of enhanced migration of these cells to the CNS of Dox‐treated double tg mice when compared to single tg mice. Molecules that neutrophils are known to possess that potentially could impact cell survival include neutrophil elastase, cathepsin G, and matrix metalloproteinases 39. Indeed, neutrophils have clearly been shown to exacerbate brain injury in a model of focal cerebral ischemia 47. Blocking neutrophil trafficking to the CNS or inhibition of the NAPDH complex in mouse models of ischemic injury results in a dramatic reduction in both the vascular endothelium and brain parenchyma 47, 48. Moreover, both the generation of ROS and nitric oxide is thought to contribute to vascular damage as well as impact axonal integrity and oligodendrocyte viability 49, 50. Neutrophils are also implicated in exacerbating lesion development within spinal cords on patients with neuromyelitis optica (NMO) 51, and inhibition of neutrophil elastase, a serine protease released from the primary granules of neutrophils, within mouse models of NMO resulted in reduced neuroinflammation and myelin loss 52, 53. Within the EAE model of chronic neurologic disease, neutrophils have recently been reported to have a role in maturating local APCs within the CNS thus potentially contributing to increased disease severity by increasing numbers of autoreactive T cells 54. However, we believe it unlikely that the increase in disease severity is the result of enhanced macrophages presenting antigen as we determined there was no increase in either activated macrophage or microglia in the CNS of Dox‐treated double tg mice nor were there increases in either Th1 and/or Th17 CD4^+^ T‐cell responses.

The importance of neutrophils in enhancing white matter damage in Dox‐treated double tg mice was emphasized by demonstrating that blocking neutrophil accumulation within the CNS via anti‐CXCR2 treatment dramatically reduced demyelination. Previous studies from our laboratory 30, 32 and others 9, 47, 55, 56 have demonstrated that administration of anti‐CXCR2 blocking antibody limits neutrophil mobilization from the bone marrow and limits migration to the CNS in various models of neurodegeneration. Our findings provide further evidence that neutrophils augment neurologic disease and emphasize that therapies targeting neutrophil accumulation within the CNS may offer novel alternative therapies for treating neuroinflammatory diseases.

## Materials and methods

### Mice

pBI‐CXCL1‐rtTA double transgenic mice (developed on the C57BL/6 background) were developed as previously described 21. In brief, pBI‐CXCL1 transgenic mice were generated by the University of California, Irvine transgenic mouse facility through DNA microinjection of fertilized C56BL/6 eggs using the linearized pBI‐CXCL1 construct 57. The five resulting founder transgenic (tg) mice were mated to WT C57BL/6 mice to identify F1 offspring containing the transgene. To generate double transgenic (tg) mice, hemizygous pBI‐CXCL1 transgenics were crossed to hemizygous GFAP‐rtTA*M2 mice (JAX), resulting in double transgenic mice (pBI‐CXCL1‐rtTA), single tg (rtTA‐GFAP or pBI‐CXCL1) or WT. Dox administration to double tg results in elevated CXCL1 transgene expression within astrocytes corresponding with increased CXCL1 protein and neutrophil accumulation whereas Dox treatment of single tg had no effect on CXCL1 expression nor neutrophil accumulation within the CNS 21.

### MOG_35–55_ EAE immunization

For induction of EAE, experimental mice (aged 6–8 weeks old) were injected subcutaneously in the flanks with 200 μL of 1 mM MOG_35–55_ peptide (DNA/Peptide Synthesis Core Facility, University of Utah) emulsified with reconstituted complete Freund's adjuvant (Pierce Biotechnology, Waltham, MA, USA) containing *Mycobacterium tuberculosis* H37 Ra (2 mg/mL) (Difco Laboratories, Franklin Lakes, NJ, USA). Mice were injected intravenously with 100 μL *Bordetella pertussis* toxin (Ptx) (List Biological Laboratories Inc., Campbell, CA, USA) at 0.2 μg/mouse on days 0 and 2 following sensitization. Double and single transgenic animals were injected with doxycycline (Dox) (50 mg/kg via i.p. injection) starting at day 9 following EAE induction and continuing through day 19 postimmunization (p.i.). Experimental mice injected with MOG_35–55_ were scored daily for clinical signs through day 21 following immunization using previously described methods 58. In brief, 0, normal mouse with no signs of disease; 1, limp tail or hind‐limb weakness; 2, limp tail and hind‐limb weakness; 3, partial hind‐limb paralysis; 4, complete hind‐limb paralysis; 5, moribund state/death. All animal experiments were approved by the University of Utah Institutional Animal Care and Use Committee protocol 18–01005.

### Flow cytometry

Inflammatory leukocytes infiltrating into the CNS were isolated using an established protocol (3,4). In short, CNS tissue was minced and leukocytes were isolated using a two‐step Percoll gradient (90% and 63%). The isolated cells were collected and then washed prior to staining. Cells were incubated in an anti‐CD16/32 Fc Block (BD Biosciences, San Jose, CA) at a dilution of 1:200. Cells were stained with fluorescently tagged rat anti‐mouse IgG for the following cell surface antibodies, Ly6G FITC (1A8), CD11b PE (M1/70), CD45 BV510 (30‐F11), CD8 PE‐Cy7 (53–6.7), (BD Biosciences), Ly6C APC (HK1.4), CD4 BV785 (RM4‐5), F4/80 FITC (BM8), I‐A/I‐E APC (M5/114.15.2), CD11c FITC (N418) (Biolegend, San Diego, CA), and CD4 FITC (RM4‐5) or Armenian hamster anti‐mouse IgG for CD80 PerCP‐Cy5.5 (16‐10A1) (BD Biosciences). A full description of gating strategies for flow cytometric staining and intracellular cytokine staining is provided in the Supporting Information Figs. S1–S4.

### Ex vivo peptide restimulation

Spinal Isolated spinal cord leukocytes that were isolated using a Percoll gradient as above, were stimulated with MOG_35–55_ peptide or 50 ng/mL PMA (Sigma, St. Louis, MO) with 2 ug/mL Ionomycin (Sigma, St. Louis, MO) and BD Cytofix/Cytoperm Plus Fixation/Permeabilization Kit (with BD GolgiPlug protein transport inhibitor containing brefeldin A) (BD Biosciences, San Jose, CA) and incubated at 37°C for 6 hours or 5 hours, respectively. Cells were stained the surface marker CD4 (eBiosciences, San Diego, CA) and for intracellular cytokines including Rat anti‐mouse IgG, IL‐17A (eBiosciences, San Diego, CA) and IFN‐γ (BD Biosciences).

### Quantitative real‐time PCR

Total cDNA from brains and spinal cords of sham and EAE immunized mice at day 12 following disease induction was generated via Superscript III (Life Tech., Carlsbad, CA) following homogenization in Trizol (Life Tech., Carlsbad, CA).

Expression of mRNA from defined mouse chemokine and cytokine genes including *Cxcl1, Cxcl2, Cxcl10, Ccl2, Ccl5, IFN‐γ*, and *IL‐17A* within the brain of experimental mice was determined by qPCR using SYBR Green I Master (Roche) on a LightCycler 480 (Roche) and normalized to β‐actin 59.

### Histology

Mice were euthanized according to IACUC guidelines and spinal cords were removed, fixed overnight in 4% paraformaldehyde (PFA) at 4°C and separated into eight 1.5 mm sections. Each section was cryoprotected in 30% sucrose for five days before embedding in OCT; 8 μM thick coronal sections were cut and stained with luxol fast blue (LFB) combined with H&E. Pathology scoring of spinal cord sections was performed using previously described methods; a minimum number of three mice/group with 6–8 section/mouse assessed 60. In brief, for scoring of spinal cord section, each spinal cord section was divided into four quadrants (the ventral funiculus, the dorsal funiculus, and each lateral funiculus) 60; any quadrant containing demyelination, meningeal infiltration, or perivascular cuffing was given a score of 1 in that pathological class. The total number of positive quadrants for each pathological class was determined and then divided by the total number of quadrants present on the slide and multiplied by 100 to give the percent involvement for each pathological class.

### Immunofluorescence

Spinal cord sections were processed as described above. For immunofluorescence, slides were first desiccated for two hours and blocked with 5% Normal Donkey Serum with or without 0.3% Triton‐X 100. Primary antibodies were incubated overnight at 4°C: goat anti‐CXCL1 1:50 (R&D Systems, Minneapolis, MN), rabbit anti‐GFAP 1:500 (Life Technologies, Carlsbad, CA), and rat anti‐Ly6B.2 1:100 (Serotec, Raleigh, NC). Images were analyzed using the Image J software (NIH) according to previously described methods 61, 62. Quantification of Ly6B.2‐positive cells within spinal cords of experimental mice was determined by counting cells in a minimum of five spinal cord sections/per mouse with a minimum of three mice per group.

### Antibody administration

Rabbit polyclonal antiserum was generated to a 17‐amino acid portion of the amino‐terminus ligand‐binding domain of specific for mouse CXCR2 (MGEFKVDKFNIEDFFSG) (Cocalico Biologicals, Inc., Stevens, PA) and used to target neutrophils as previously described has been previously described 9, 32, 55. Normal rabbit serum (NRS) from preimmunized rabbits was used as controls. Experimental mice were injected i.p. with 0.5 mL with either anti‐CXCR2 or NRS every 2–3 days starting on day 9 following EAE induction and ending on or before day 19.

### Statistical analysis

Flow cytometric data were analyzed with FlowJo (Tree Star Inc.) and an unpaired Student's *t*‐test was used, with a *p* < 0.05 being considered as significant.

## Conflict of interest

The authors declare no commercial or financial conflict of interest.

**Abbreviations**
Dox: doxycycline
GFAP: glial fibrillary acidic protein
JHMV: JHM strain of mouse hepatitis virus
rtTA: reverse tetracycline‐controlled trans activator

## Supporting information

Peer review correspondence

Supporting Information Figures S1–S5
