# Supplementary material for: Induced CNS expression of CXCL1 augments neurologic disease in a murine model of multiple sclerosis via enhanced neutrophil recruitment
Source: Eur J Immunol. 2018 May 16;48(7):1199–210. doi: 10.1002/eji.201747442 (PMC6033633; doi:10.1002/eji.201747442)
Supplement: Supplementary file 2 — Supporting Information Figures S1–S5 [file EJI-48-1199-s002.pdf]

European Journal of Immunology

Supporting Information
for
DOI 10.1002/eji.201747442

Jonathan J. Grist, Brett S. Marro, Dominic D. Skinner, Amber R. Syage, Colleen Worne, Daniel J. Doty, Robert S. Fujinami and Thomas E. Lane

Induced CNS expression of CXCL1 augments neurologic disease in a murine model of multiple sclerosis via enhanced neutrophil recruitment

© 2018 The Authors. *European Journal of Immunology* published by WILEY-VCH Verlag GmbH & Co. KGaA, Weinheim.

Supplemental Figure 1

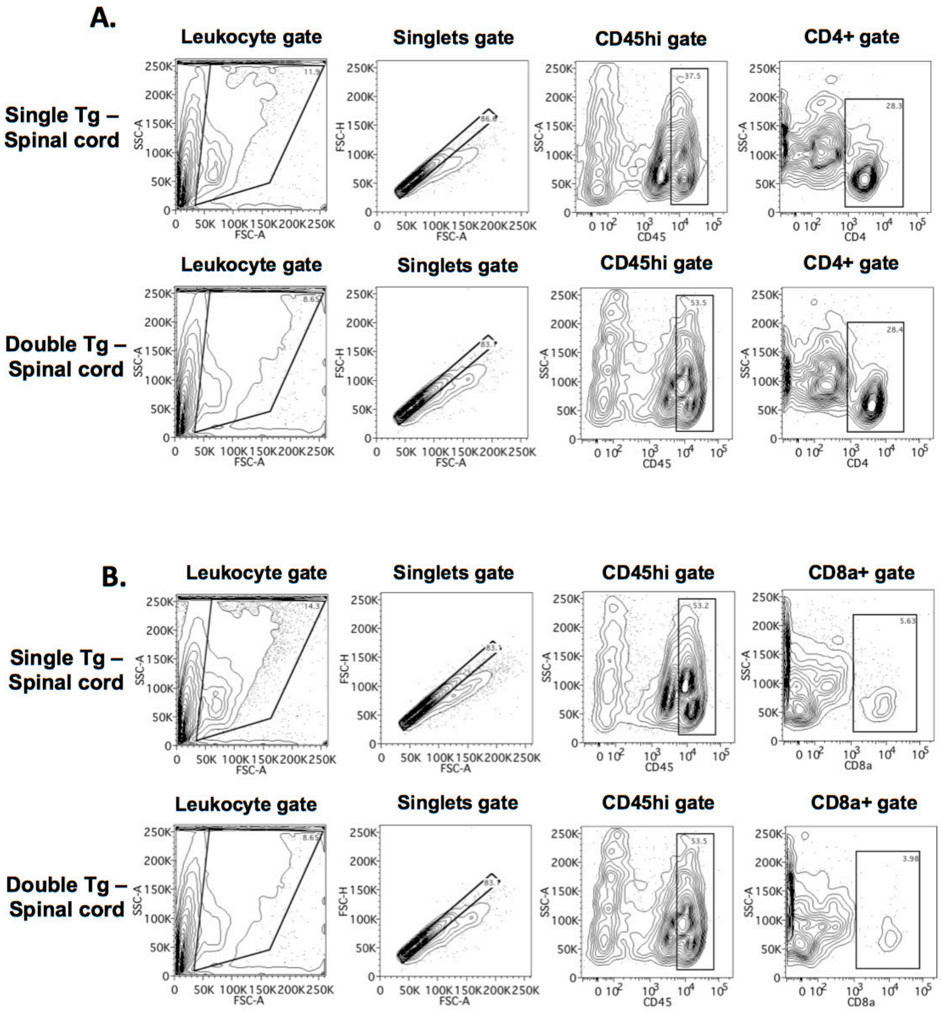

Supplemental Figure 1. Contour plots showing the gating scheme for identification of (A) CD45+CD4+ and (B) CD45+CD8a+ T cells isolated from the spinal cords of single and double tg mice. The final contour plots on the right show the CD4+ and CD8a+ T cells that were analyzed as indicated by the black rectangular gate.

Supplemental Figure 2

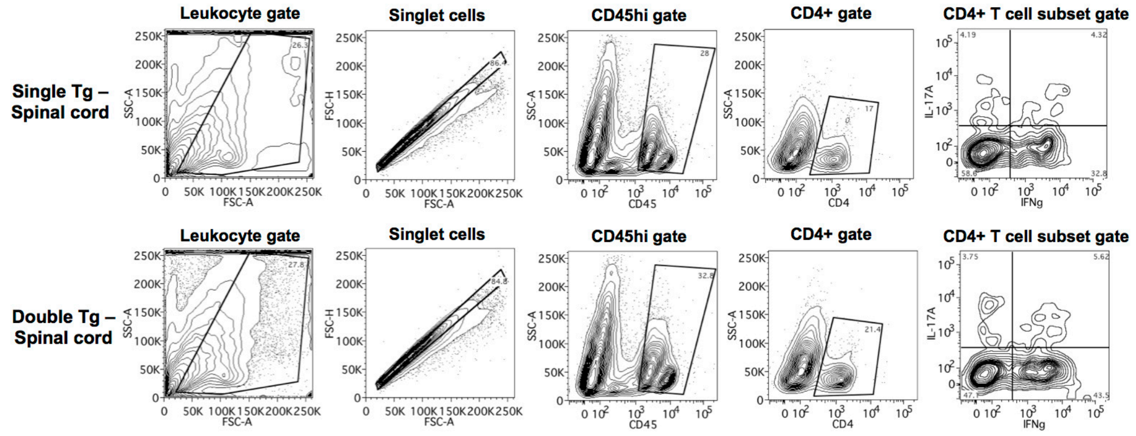

Supplemental Figure 2. Contour plots depicting the gating scheme for isolating CD45+CD4+ T cells from the spinal cords of single and double tg mice and subsequent intracellular staining for IL-17A and IFN-γ. The final contour plots on the right indicate CD45+CD4+ T cells expressing either IL-17A, IFN-γ or dual-positive for IL-17A/IFN-γ.

Supplemental Figure 3

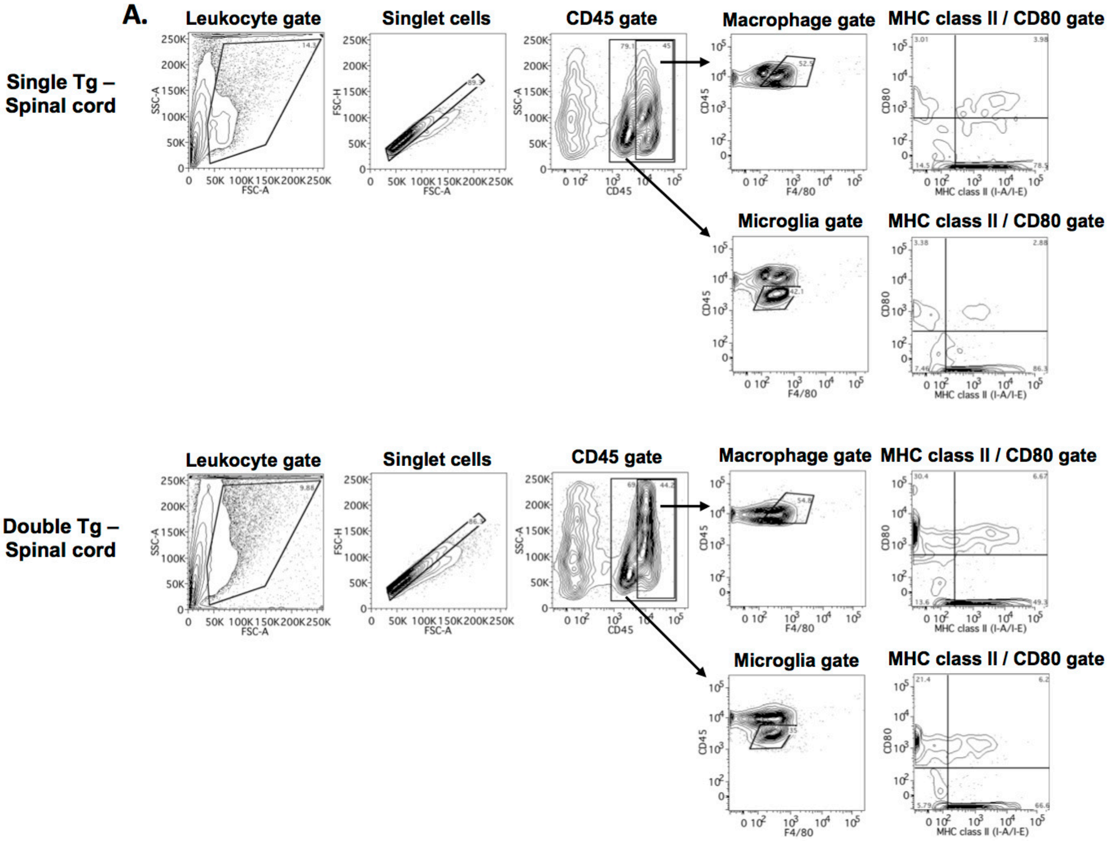

Supplemental Figure 3. Contour blots depicting the gating scheme for identification of macrophages (CD45+F480^{hi}) and microglia (CD45+F480^{lo}) from spinal cords of single tg and double tg mice. Gated populations were subsequently stained for MHC class II and CD80. The final contour plots on the right show to identify dual-positive cells in the upper-right quadrant.

Supplemental Figure 4

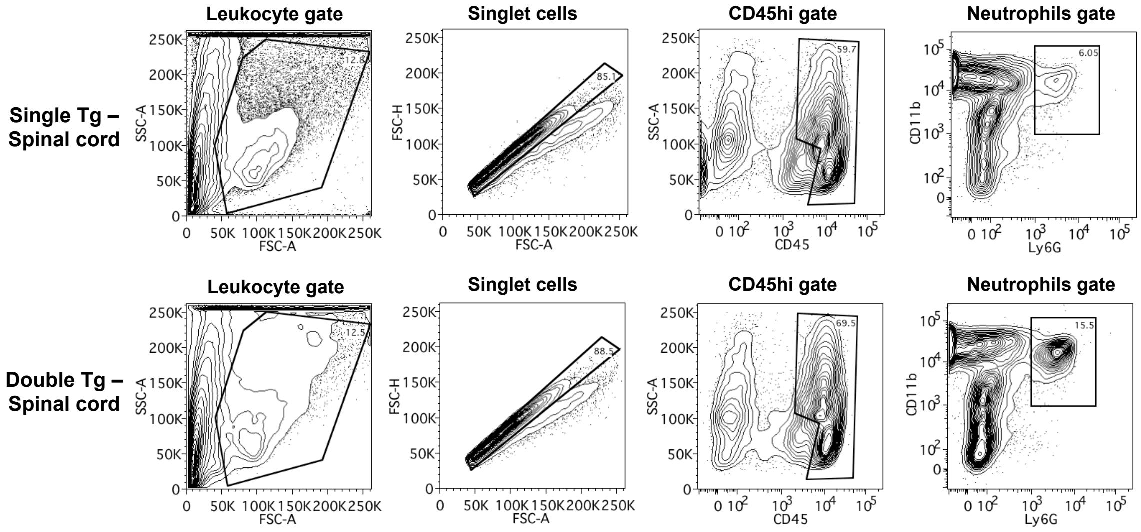

Supplemental Figure 4. Contour plots representing gating scheme employed in identifying CD11b+Ly6G+ neutrophils isolated from the spinal cords of single tg and double tg mice. The final contour plots on the right reveal the neutrophils that were analyzed as indicated by the black rectangular gate.

Supplemental Figure 5

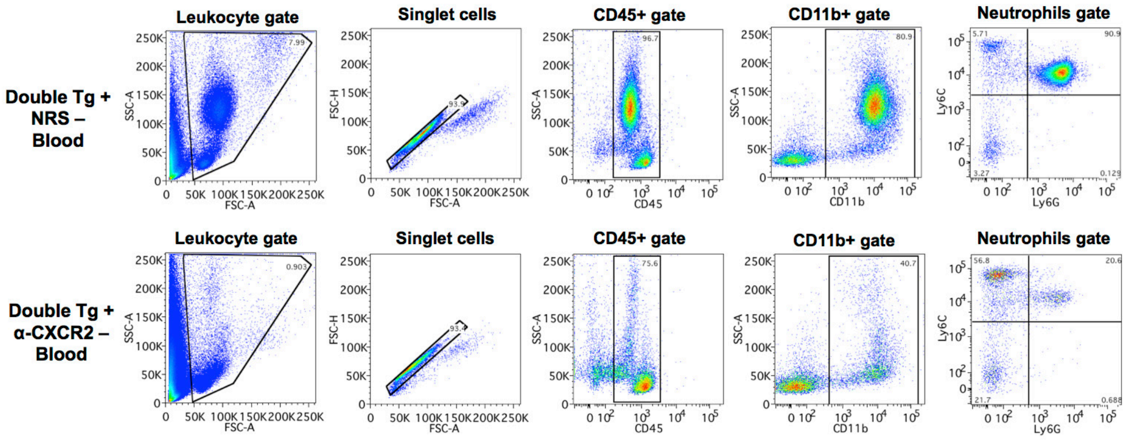

Supplemental Figure 5. Flow plots representing gating scheme employed in identifying CD11b+Ly6G+ neutrophils isolated from the blood of double tg mice treated with either NRS or anti-CXCR2 antibody. The final contour plots on the right reveal the neutrophils that were analyzed as indicated by the black rectangular gate.
