# Supplementary material for: Induced CNS expression of CXCL1 augments neurologic disease in a murine model of multiple sclerosis via enhanced neutrophil recruitment
Source: Eur J Immunol. 2018 May 16;48(7):1199–210. doi: 10.1002/eji.201747442 (PMC6033633; doi:10.1002/eji.201747442)
Supplement: Supplementary file 1 — Peer review correspondence [file EJI-48-1199-s001.pdf]

European Journal of Immunology

Induced CNS expression of CXCL1 augments neurologic disease in a murine model of multiple sclerosis via enhanced neutrophil recruitment

Jonathan J. Grist1, Brett S. Marro, Dominic D. Skinner, Amber R. Syage, Colleen Worne, Daniel J. Doty, Robert S. Fujinami and Thomas E. Lane

Correspondence: Dr. Thomas E. Lane, Department of Pathology, Division of Microbiology & Immunology, University of Utah, School of Medicine, Salt Lake City, Utah, United States

---

Review Timeline:	Submission date:	04-Dec-2017
	First Editorial decision:	09-Jan-2018
	Revision received:	17-Feb-2018
	Accepted:	20-Apr-2018

Handling Executive Committee member: Prof. Britta Engelhardt

Please note that the correspondence below does not include the standard editorial instructions regarding preparation and submission of revised manuscripts, only the scientific revisions requested and addressed.

First Editorial Decision
09-Jan-2018

Dear Dr. Lane,

Manuscript ID eji.201747442 entitled "Induced central nervous system expression of CXCL1 augments neurologic disease in a model of multiple sclerosis via enhanced neutrophil recruitment" which you submitted to the European Journal of Immunology has been reviewed. Please accept our apologies for the delay in the decision that was due to holiday season and unavailability of the Executive Committee members. The comments of the referees are included at the bottom of this letter.

We encourage you to take into account the comments of the referees and to reply to their critiques in detail.

A revised version of your manuscript that takes into account the comments of the referees will be reconsidered for publication. Should you disagree with any of the referees concerns, you should address this in your point-by-point response and provide solid scientific reasons for why you will not make the requested changes.

You should also pay close attention to the editorial comments included below. **In particular, please edit your figure legends to follow Journal standards as outlined in the editorial comments. For all data, please report the number of independent experiments and number of samples per experiment (or experimental replicates). For flow cytometry data please show the full gating strategy and report all the fluorochromes used. Please see detailed instructions below. Failure to do this will result in delays in the re-review process.**

Please note that submitting a revision of your manuscript does not guarantee eventual acceptance, and that your revision will be re-reviewed by the referees before a decision is rendered.

If the revision of the paper is expected to take more than three months, please inform the editorial office. Revisions taking longer than six months may be assessed by new referees to ensure the relevance and timeliness of the data.

Once again, thank you for submitting your manuscript to European Journal of Immunology and we look forward to receiving your revision.

Yours sincerely,
Marta Vuerich

On behalf of
Prof. Britta Engelhardt

Dr. Marta Vuerich
Editorial Office
European Journal of Immunology
e-mail: ejied@wiley.com
www.eji-journal.eu

****

Reviewer: 1

Comments to the Author
In this study, the authors have examined how transgenic expression of CXCL1 in astrocytes affects EAE. They conclude that CXCL1 overexpression increases EAE severity by attracting neutrophils into the CNS.

The scope of this study is limited for the following reasons:

The conclusion is not original: the effect of CXCL1 is already well known. This study represents only a minor advance for the field.

In EAE, CXCL1 is mainly expressed by endothelial cells and probably some leukocytes. However, the authors have chosen to ectopically express CXCL1 in astrocytes. Thus, the physiological relevance of this study is unclear.

The authors have validated the role of neutrophils by administration of anti-CXCR2 antibody. This approach has previously been used by another group in the same model and the results are not original.

The background section is not well written and does not accurately represent the state of the field. For example, the study by Roy et al. on the expression and role of CXCL1 in EAE has been ignored. After a focused review of the literature, the authors should have formulated an original question and hypothesis.

The study is relatively small. Figures 2-4 could have been merged. Other analyses could have been done to address an original question.

Reviewer: 2

Comments to the Author
The study demonstrates that overexpression of chemokine CXCL1 in the CNS (specifically in astrocytes) drives worsened EAE that is neutrophil-specific. The story is straightforward and the data are convincing. My recommendation is "Accept with minor alterations". However, I would like to see one additional piece of data (below, point #2):

1) p5, line 34: CXCL1 is a chemokine, not a cytokine;

2) Given the discrepancy with the earlier Omari study, I would like to see evidence, in unimmunized, DOX-treated double-Tg mice, that CXCL1 expression is increased in the CNS. If the data from 1D-F are indeed from unimmunized mice, this needs to be stated more clearly.

3) While it has been previously described, the Pathology Score used in 5E needs to be at least briefly described here in the Materials and Methods.

First Revision – authors' response

17-Feb-2018

Reviewer 1:
1) The conclusion is not original: the effect of CXCL1 is already well known. This study represents only a minor advance for the field. We recognize the reviewers concern. However, we would argue that the role of CXCL1 has largely been considered during early pre-clinical stages of EAE in which a role for attracting neutrophils was important in subsequent immune cell infiltration (Carlson et al., JEM 2008). How chronic expression of CXCL1 affects clinical disease and demyelination has not been extensively studied. Raine and colleagues (Omari et al., Am J Pathol, 2009) published that inducible expression of CXCL1 from astrocytes resulted in neuroprotection and remyelination following induction of MOG-induced EAE. In marked contrast, the findings reported in our study show that in a MOG-induced EAE, overexpression of CXCL1 results in increased clinical disease and demyelination associated with increased neutrophil infiltration. As indicated in our report, the difference between our study and the AJP study is most likely that the CXCL1 transgene we employed lacked the 3’UTR region which would increase the half-life of the mRNA transcript whereas Omari et al. generated transgenic animals in which the 3’UTR remained and would have shortened the half-life due to targeted destruction. More importantly, our findings contribute to a growing number of studies that indicate an important role for myeloid cells e.g. neutrophils in enhancing white matter damage and supports targeting these cells in muting neuroinflammation and demyelination in MS patients.

2) In EAE, CXCL1 is mainly expressed by endothelial cells and probably some leukocytes. However, the authors have chosen to ectopically express CXCL1 in astrocytes. Thus, the physiological relevance of this study is unclear. The reviewer is correct in that a number of resident CNS cells as well as inflammatory cells express CXCL1 under inflammatory disease conditions including EAE. Our rationale for selecting astrocytes for expression of CXCL1 include previous EAE studies reporting that these cells secrete CXCL1 (Glabinski et al., Am. J. Pathol., 1997; Luo et al., J. Immunol., 2000) and that ectopic expression in astrocytes modulates the severity of EAE (Omari et al., Am. J. Pathol., 2009). In addition, CXCL1 is expressed by reactive astrocytes in MS lesions as well as cultured astrocytes in response to treatment with IL-1beta (Omari et al., Glia, 2006). We would also point out that in mouse models of viral-induced encephalomyelitis and demyelination, astrocytes were a predominant source of CXCL1 (Hosking et al., Plos Pathog., 2009; Hosking et al., PLoS One, 2010; Rubio and Sanz-Rodriguez, Virology, 2007). Collectively, these studies formed the basis for selecting astrocytes as the target cell for inducible expression of CXCL1. This is explained in the revised manuscript.

3) The background section is not well written and does not accurately represent the state of the field. For example, the study by Roy et al. on the expression and role of CXCL1 in EAE has been ignored. After a focused review of the literature, the authors should have formulated an original question and hypothesis. We’ve taken the reviewer’s comments seriously and have modified the Background section to better explain our rationale for examining the role of CXCL1 in contributing to both clinical disease and demyelination. In addition, we apologize for the oversight regarding the Roy et al., J. Neuroinflammation, 2012 and this is now included in the revised article. Indeed, our findings support the findings reported in this paper that CXCL1 does contribute to clinical EAE and further extend these findings by clearly

demonstrating that CXCL1-mediated attraction of neutrophils amplify the severity of demyelination.

4) The study is relatively small. Figures 2-4 could have been merged. Other analyses could have been done to address an original question. We respectfully disagree with the reviewer regarding his/her opinions and have kept the figures the same.

Reviewer 2:
1) p5, line 34: CXCL1 is a chemokine, not a cytokine: This has now been changed.

2) Given the discrepancy with the earlier Omari study, I would like to see evidence, in unimmunized, DOX-treated double-Tg mice, that CXCL1 expression is increased in the CNS. If the data from 1D-F are indeed from unimmunized mice, this needs to be stated more clearly. This has been performed and the results are presented within the revised Figure 1E. In brief, we report increased levels of CXCL1 within the spinal cords of Dox-treated mice in the absence of EAE induction. These findings argue that Dox-induced CXCL1 within the CNS occurs independent of experimentally-induced neuroinflammatory disease.

3) While it has been previously described, the Pathology Score used in 5E needs to be at least briefly described here in the Materials and Methods. This has now been included in the revised Materials and Methods section.

Second Editorial Decision

26-Mar-2018

Dear Dr. Lane,

It is a pleasure to provisionally accept your manuscript entitled "Induced central nervous system expression of CXCL1 augments neurologic disease in a model of multiple sclerosis via enhanced neutrophil recruitment" for publication in the European Journal of Immunology. For final acceptance, please follow the instructions below and return the requested items as soon as possible as we cannot process your manuscript further until all items listed below are dealt with.

Please note that EJI articles are now published online a few days after final acceptance (see Accepted Articles: https://onlinelibrary.wiley.com/toc/15214141/0/ja). The files used for the Accepted Articles are the final files and information supplied by you in Manuscript Central. You should therefore check that all the information (including author names) is correct as changes will NOT be permitted until the proofs stage.

European Journal of Immunology

We look forward to hearing from you and thank you for submitting your manuscript to the European Journal of Immunology.

Yours sincerely,
Nadja Bakocevic

on behalf of
Prof. Britta Engelhardt

Dr. Nadja Bakocevic
Editorial Office
European Journal of Immunology
e-mail: ejied@wiley.com
www.eji-journal.eu
